# Best marketing strategy selection using fractional factorial design with analytic hierarchy process

**DOI:** 10.1016/j.mex.2020.100927

**Published:** 2020-05-23

**Authors:** Anas Al-Dawalibi, Ibrahim H. Al-Dali, Bandar A. Alkhayyal

**Affiliations:** a Department of Systems Engineering, College of Computer Sciences and Engineering, King Fahd University of Petroleum and Minerals, Dhahran, Saudi Arabia; b Engineering Management Department, College of Engineering, Prince Sultan university, Riyadh, Saudi Arabia

**Keywords:** Screening Experiment, Marketing Strategies Selection, Fractional Factorial Design, DOE, AHP, MCDM, RSM

## Abstract

Marketing strategies selection for the purpose of increasing the sales revenue is an essential problem that can face any marketing team. Many companies facing difficulties of identifying the best marketing strategy for certain product or service, because the marketing plan involves a large number of strategies (factors) that can affect the sales revenue response. $n^{k-p}$Fractional Factorials Design has been used as screening experiment to identify the most important factors which have a main effect to response the (sales revenue). Then further decision-making technique has been introduced to compare the criteria of selected strategies (factors) using analytic hierarchy process technique (AHP). Basically, AHP will provides a comprehensive and rational framework for structuring decisions criterial to each main factor where it provides the best factor or marketing strategy that can increase the sales revenue for the company**.**

In which, the suggested methodology aims to enable decision maker the following:

• The opportunity to investigate a large number of marketing strategies with minimum experiments run and to enable the decision maker to identify which strategies have significant effect on response.
• Select the best marketing strategy that maximize the targeted response (sales revenue)

## Graphical abstract

**Figure:**
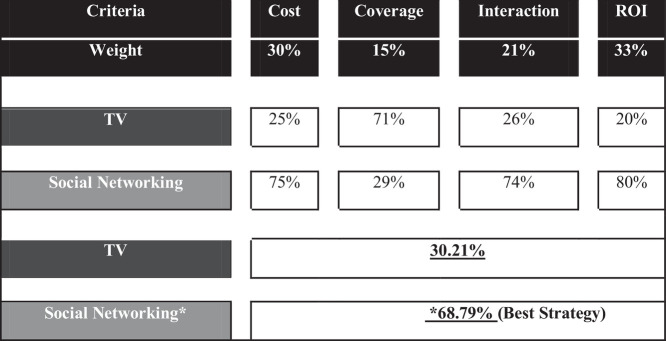

Specifications Table

**Table:** 

Subject Area	Engineering
More specific subject area	Selecting the best marketing strategies using statistical and multi-criterial-descion-making tools
Method name	Best marketing strategies selection
Name and reference of original method	Design of experiment (DOE), Fractional factorial design (FFD), and method of steeps ascent
Resource availability	Minitab and Excel

## Introduction & background

Applying the experimental design can provide potential value for different types of applications, in which it can help companies or organizations to maximize their profits and minimize their costs [1,4]. Moreover, the design of experiment method has been commonly used in the area of manufacturing but rarely used in the service industry. In which, a two-level fractional factorial design of 16 runs is used by [6] to increase the direct mail response of Mother Jones Magazine which has 7 factors influencing its response. This is in complete contrast to the traditional method where factors are changed only one at a time. In fact, fractional factorial design has been used as research tools in numerous fields such as: chemistry, electrical [2], social networks [14] and marketing [3].

Additionally, in the literature there are many different applications have been used AHP in order to provide the researchers the opportunity to compare among different decisions strategies. These applications included but not limited to: examines the buying behavior of potential furniture buyers [11], selection of the natural fibers for composite materials [10], and selection of appropriate channels of marketing. However, in theses researches there are different quantitative and qualitative characteristics have been studies using AHP to enable decision maker of selecting the best alternative strategy.

This research is more concerned with marketing strategies selection and in the line with this, the marketing literature indicates that a firm's marketing strategies effect the its market place and the firm performance through implementation of specifics pattern of resources planning to achieve marketing objective in a target market [5,8]. This implementations provide perspective suggests that goal setting and marketing strategy development systems are used as future-oriented decision-making frameworks to identify goals and select marketing strategy options that may enable these goals to be accomplished, followed by a period of enactment in which firms seek to customize the intended marketing strategy decisions to achieve the required goals [7,^8^,13] . Basically, in this study it suggested a methodology that combining design of experiment (DOE) with multi criterial decision process (AHP), in order to provides the marketing researchers, the opportunity to investigate a large number of factors or marketing strategies with minimum experiments run and to enable them to identify which strategies have significant effect on response (sales revenue).

The objective is to illustrate the use of the fraction factorial design with AHP in a marketing problems framework, in which enables decision maker to select the best marketing strategy that maximize the targeted response (sales revenue). Specific marketing strategies for a lubricant company will be introduced as illustrated example.

The remaining of this paper is organized as following: Proposed methodology for sleeting the best market strategy in Section 2. Afterwards, illustrated example will be discussed in Section 3. Then results and discussion in Section 4. Finally, we summarize and conclude in section 5.

## Proposed methodology for selecting the best marketing strategies

Any marketing plan will involve a large number of possible marketing strategies, companies are interested to select the best of these strategies that has a significant effect on the sales revenue (response). However, most commonly these companies do not have enough resources to run the large number of experiments that generated for the different marketing strategies. The objective is to use the fractional factorial design as screening experiment to determine the most effective factors (strategies) from the different available marketing strategies. Additionally, Response Surface Methodology (RSM) will be used to provide useful analysis of problem after identifying the variables (marketing strategies) that influence the sales revenue.

**Fig. 1:**
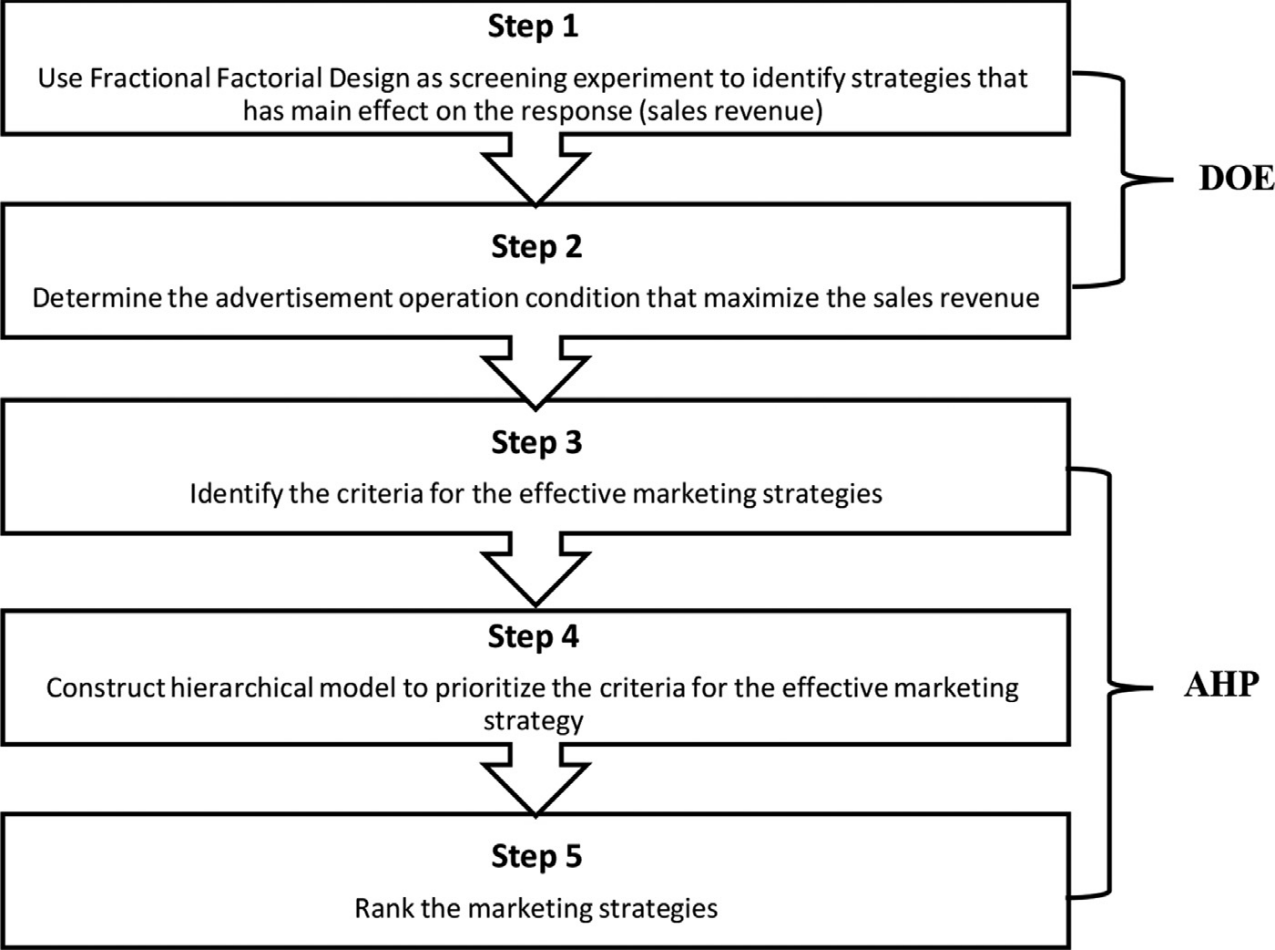
The proposed methodology to select the best marketing strategy.

After finding the most important factors that have effect on the response (Sales Revenue), an AHP technique will be used to select the best market strategy. The idea here is to find which factors or strategies have significant effect on sales revenue response, where AHP will be used to compare those strategies that have the main effect on the targeted response (Sales Revenue). Fig. 1, shows the proposed methodology to select the best marketing strategy.

### Advantage of the fractional factorial design in marketing problems

As it mentioned DOE has been used as a valuable tool for researches in different types of applications, where these applications are experimental applications in which a fixed number of levels *n* are selected for each *k* factors. For the complete DOE experiments, it required to run all combinations of levels of all factors. Intuitively, the complete DOE will consist of *n^k^* experimental runs.

According to [3] marketing problem in general involves a large number of marketing strategies that can affect the sales revenue. However, running a full factorial experiment for a marketing problem is challenging, because most companies do not have enough resources to perform *n^k^* experimental runs, as it will be too costly for the company and it needs a lot of manpower to do a full factorial experiment using only two levels of each factor. Accordingly, this study aims to take the advantage of the major use of the Fractional Factorial Design which is working as screening experiments. Where the objective is to identify those strategies (if any) that have significant effects on the response (sales revenue).

Table 1 shows how fractional factorial designs is developed. Considering a situation that involved three factors, each at two level, but this particular experiment cannot afford running the full factorial experiment $2^{3}=8$ treatment combinations. Thus, it decided to use on-half fraction factorial design of 2^3,^ because the new design will consist of $2^{3-1}=4$ treatment combination instead of 8, and because of that, it was called on-half fraction factorial design.

**Table 1:** The two one-half fractions of the 2^3^ design.

Run	$2^{3-1},The\;principal\;fraction\;I=ABC$			Response
	*A*	*B*	C=AB	
1	−	−	+	*y*_1_
2	+	−	−	*y*_2_
3	−	${+}^{\prime }$	−	*y*_3_
4	+	+	+	*y*_4_

Estimating the main effects for all factor can be calculated by using Table 1. Where this table has be obtained by indicating the signs under the factors *A* and *B*. Where at each experimental run (+) indicated the factor at the high level and (−) indicate that the factor at the low level. On the other hand, the signs for the interaction columnC=AB it can be found by multiply signs for *A* and *B* columns. The main effect for each factor can be obtain by multiply each response by the related sign. For example, the estimated main effect of the factor *B* is:

(1) $$B=\frac{1}{2}\times \left(-y_{1}-y_{2}+y_{3}+y_{4}\right)\;$$

### Advantage of response surface methodology (RSM) in marketing problems

RSM is mathematical and statistical techniques that can provides useful analysis in problems. This study aims to use the RSM techniques in marketing problem in which the targeted response (sales revenue) influenced by selected marketing strategies that has been identified form the screening experiment. The ultimate objective is to find optimal conditions for the selected marketing strategies that maximize sales revenue. Using the method of steeps ascent will allow moving sequentially in the correct direction that maximize our response (sales revenue). The fitted first-order model is:

(2) $$y=\;\beta_{0}+\;\sum_{i=1}^{k}\beta_{i}.x_{i},where\;the\;parameter\;\beta_{0}\;is\;estimated\;by\;the\;average\;of\;all\;responses\;$$

According to ([9], P.485), to determine the coordinates of a point on the path of steepest-ascent, we need to follow the following algorithm:

1. Select a step size form one of the variables process, for example Δ*x_j_* . Where it is more preferable to selected the first variable form the variables that we know better bout, or selected the variable that has largest regression coefficient (*β_i_*).
2. Identify the steps size for the other variables:
  $$x_{i}=\frac{\beta_{i}}{\frac{\beta_{j}}{\Delta x_{j}}},\;where\;\frac{\beta_{j}}{\Delta x_{j}}=2\lambda \;i=1,\;2,\;3\;\cdots ,\;k\;i\neq j$$
3. Finally, convert Δ*x_j_* to the natural variables

### Advantages of analytic hierarchy process technique in marketing problems

After targeting factors that have the maximum effect on the response (sales revenue) using Fractional Factorial Design, AHP will take place in order to provide a useful methodology for examining the different criteria for each significant factor or strategy and select the best alternative strategy. AHP has been used in different marketing applications and helps company and decision makers for choosing the best decision [12]. Moreover, different quantitative and qualitative characteristics have been studies using AHP to enable decision maker to choose natural fibers for composite materials as more factors are considered and dependency among the factors also could influence the selection process [10].

## Illustrated example

A lubricant company marketing team facing difficulty of identifying the best marketing strategy for certain type of lubrication product, because the marketing plan involves a large number of factors that can affect the sales revenue response. The lubricant company is interested to select the best market strategy to identify the factors which have the most major effect in the sales revenue then selecting the best factor among those effect whose have a significant effect on the sales revenue. Those strategies (factors) are: TV advertisement, football league advertisement boards, social interaction, free of charge oil, radio advertisement, packaging, and size of the carton. Moreover, the company does not have enough resources to run $2^{7}=128$ experiments in different locations as it will be too costly for the company and it needs a lot of manpower to perform a full factorial experiment using only two levels of each factor. According to that it is decided that a screening experiment using fractional factorial deign will be performed in an effort to identify the more important strategies that has a significant effect on the sales revenue. Table 1 shows the levels for each factor for the screening experiment. The company decides to test the sales in 17 different locations are available to run the experiments, but 8 locations of the 17 will be selected randomly, the assigned treatment combinations indicated in Table 2. In these situations, often as many as k−1 variables will be examined using only *k* experimental runs which is 8 runs in our example.

**Table 2:** Factors and levels for the marketing experiment.

Symbol	Factor	Low Level	High Level
***A***	TV adv	No adv	Adv
***B***	Football league adv boards	No adv	Adv
***C***	Social Networking	No Interaction	Interaction
***D***	Free of charge (FOC) Oli	No FOC	FOC
***E***	Radio adv	No adv	Adv
***F***	Packaging	Traditional	Innovation
***G***	Size of the carton	12 bottle /carton	24 bottle/ carton

It can be seen that screening experiments should be useful in marketing strategy selection because most marketing situations involve a large number of factors especially when it comes for lubrication oils where lubricant company has many alternatives factors to choose from that can have a main effect in sales response. The marketing team decides to perform the screening experiment without replication, as replication will consume a huge amount of the company resources and, therefore, this problem has no estimate of experimental error.

Table 3 indicate which level will be used for each of the seven strategies to be used in each sales location. For instance, in location 1 there is no Tv advertisement, football league advertisement boards, social interaction, and size of the carton these factors are is low level(−) in location 1. One other hand, Free of charge oil, radio advertisement and packaging are in high level(+) in location 1.

**Table 3:** Levels for factors to be used in each sales area.

	Factors							Response
*Sales* *Location*	*A*	*B*	*C*	D=AB	E=AC	F=BC	G=ABC	*Sales* 1000 (*SR*)/*week*
1	−	−	−	+	+	+	−	**155**
2	+	−	−	−	−	+	+	**675**
3	−	+	−	−	+	−	+	**410**
4	+	+	−	+	−	−	−	**513**
5	−	−	+	+	−	−	+	**575**
6	+	−	+	−	+	−	−	**1050**
7	−	+	+	−	−	+	−	**750**
8	+	+	+	+	+	+	+	**1020**

## Results and discussions

Minitab has been used for data analysis and comparison to solve the problem in Table 3. And all the results have been illustrated in tables and graphs below.

### Identifying strategies that have a main effect on the sales revenue response using fractional factorial design

Table 3, shows the main effect and sum of squares (For more information see APPENDX A and APPENDEX B respectively), that has been conducted in order to evaluated the strategies that have a significant effect in the sales revenue. Strategy A (TV adv) and strategy C (Social Interaction) have a significant effect on response as percent of contribution on the response is high.

As mentioned above, in the screening experiment it has been decided to performed the experiment without replication, therefore, we have no estimated error.

**Fig. 2:**
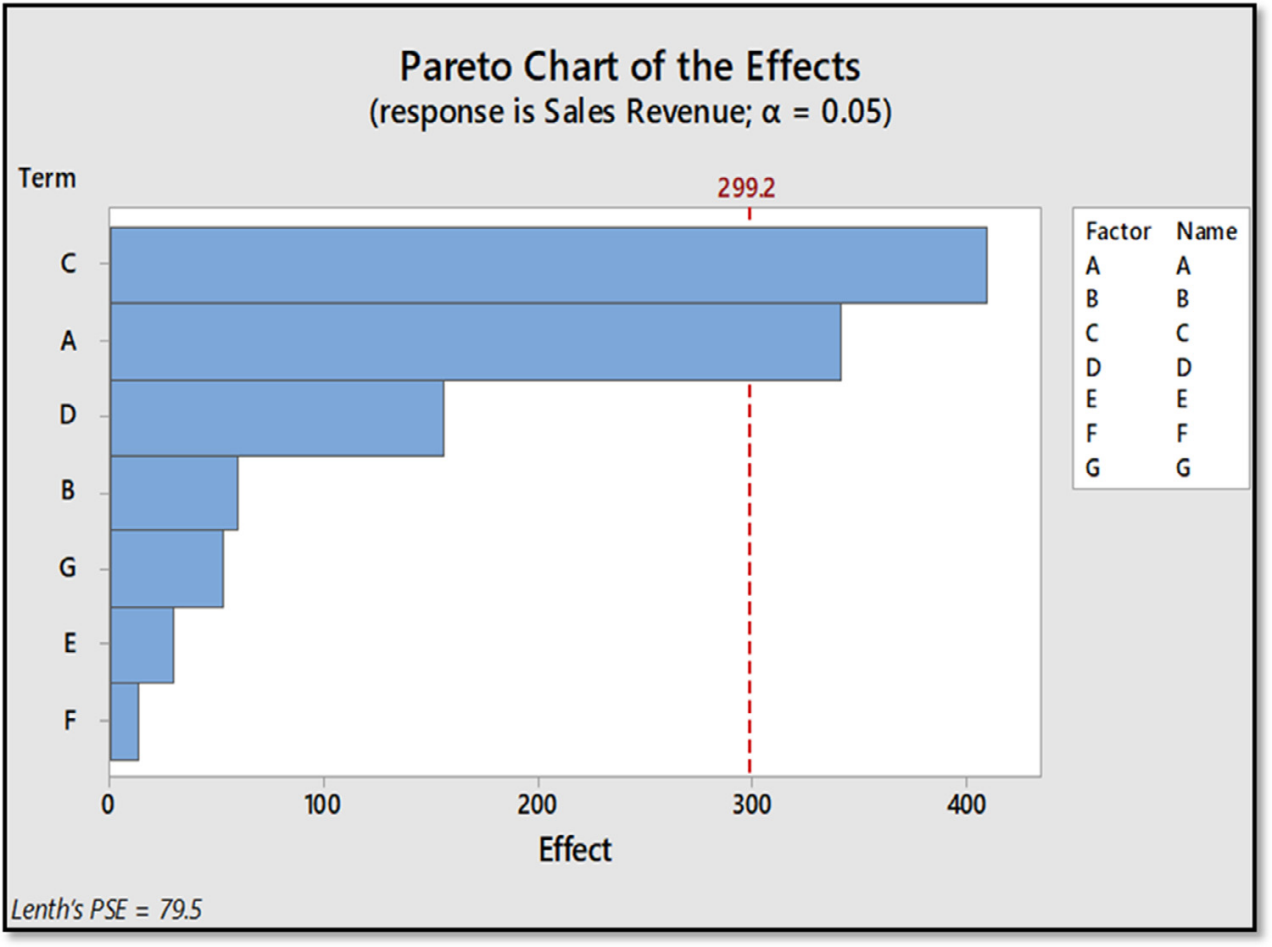
Pareto Chart.

Also, Minitab again was used in the construction of graphs and plots that illustrated the significance of specific main factors and interactions that would have an effect on the sales revenue of the company. The pareto chart was obtained as shown in Fig. 2. In which it can be found, the critical value at 0.05 level is 299.2. As it can be observed from the figure, only Factors *A* and *C* exceeded the critical value by having an effect value of 342 and 410.5 respectively.

**Fig. 3:**
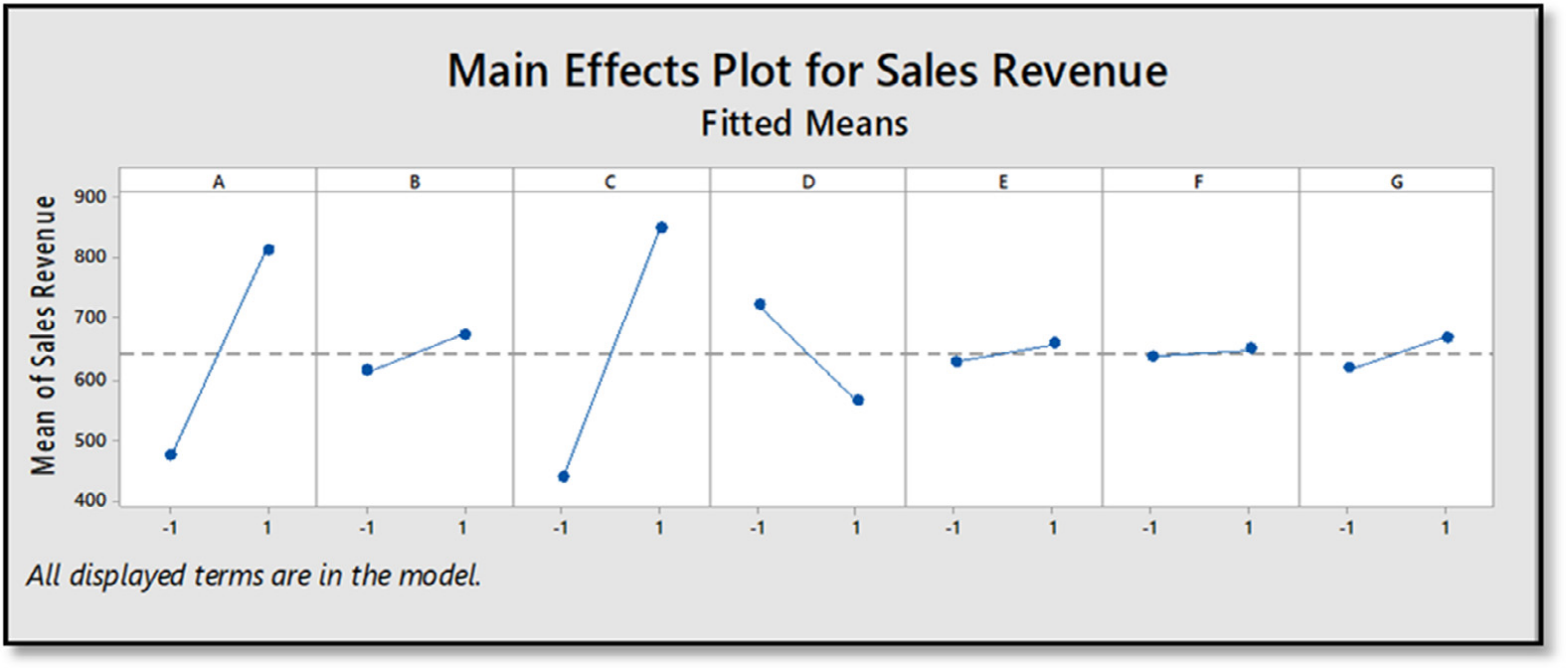
Plots showing the revenue for change in level of each factor.

Moreover, Fig. 3 illustrates the revenue when there is a change in the level of each Factor. The plot of factor *A* and factor *C* affirm the fact that they have significant positive effect on the model response whereas Factor *D* (product free of charge) has a negative response on the revenue. The reason being for this interesting observation is that providing the product free of charge will incur the company a loss and provide the customers with an incentive to fulfill their demand without having to purchase the product itself. As for the remaining plots, the rest of the plots were not having any significant effect on the response along with factor *D*.

All of this brings us to our final regression model and response surface. The regression model for predicting sales revenue is:

(3) =643.5+171A+29.75B+205.3C−77.75D+15.25E+6.5F+26.5G

The coefficient of all the factors in the regression model above can be obtained by dividing the estimated effect of each factor by 2 since we have 2 levels for each factor.

The value of 643.5 is found by taking the average of the sales column from Table 2 as shown in Eq. (4):

(4) $$\beta_{0}=\frac{\left(155\;+\;675\;+\;410\;+\;513\;+\;575\;+1050\;+\;750\;+\;1020\right)}{8}\;=\;643.5\;$$

Now, writing the regression model taking into consideration only the significant factors provides us the following with *Y* denoting the sales revenue of the problem:

(5) Y=643.5+171A+205.3C

Finally, it can be concluded that marketing strategy *A* (TV Advertisement) and marketing strategy *C* (Social Interaction) are the only factors having a true effect on the revenue from selling this product. All of the interactions of factors in addition to the remaining factors are insignificant in the context of adding a value to the company's objective of optimizing its sales revenue (Tables 4 and 5).

**Table 4:** Shows the main effect and sum of squares.

Factor	Main Effect	Sum of Squares	Percent Contribution%
***A***	342.00	233928	*36.89}
***B***	59.50	7081	1.12%
***C***	410.50	337020	*53.14}
***D***	−155.50	48361	7.63%
***E***	30.50	1860	0.29%
***F***	13.00	338	0.05%
***G***	53.00	5618	0.9%
	***SS Total***	***634206***

**Table 5:** Processed data for fitting the first order model.

Original + Steps Size	Coded Variables		Natural Variables		Sales 1000 ( {SR} )/week
	*x*_1_	*x*_2_	*A*	*C*	*y*
***Original***	0	0	80	75	
**Δ**	0.83	1	33.33	50	
Orginal+Δ	1.66	2	113.33	125	650
Orginal+2Δ	2.49	3	193.33	200	670
Orginal+3Δ	3.33	4	273.33	275	676
Orginal+4Δ	4.17	5	353.33	350	685
Orginal+5Δ	4.99	6	433.33	425	692
Orginal+6Δ	5.83	7	513.33	500	695
Orginal+7Δ	6.664	8	593.33	575	700
Orginal+8Δ	7.49	9	673.33	650	693
Orginal+9Δ	8.33	10	753.33	725	690

### Determining advertisements operation conditions that maximize sales revenue

The company marketing team is interesting in determining advertisement operation conditions that maximize the sales revenue. The two strategies that have been selected form the screening experiment in Section 4.1, they will be considered by the marketing team as controllable variables that influence the sales revenue. Accordingly, the first order model is:

$$Y=\;643.5+171x_{1}+205.3x_{2}$$

Where the coded variables *x*_1_ and *x*_2_ represent *A* (TV Advertisement) and *C* (Social Interaction) respectively, for achieving the targeted model in eq (6) the marketing team run the experiment for two factors at the following regions: for *A* Tv Advertisement (40 ≤ *A*  ≤ 120 *min*/*week*) and for *C* (Social Interaction) (50 ≤ *C* ≤ 100 *min*/*week*). Now, if the *A* denotes the natural variable the time for Tv advertisement and *C* denotes the natural variable for the time for the Social Interaction, then the coded variables are:

$$x_{1}=\;\frac{A-80}{40};\;x_{2}=\;\frac{C-75}{50}$$

Thus, we have $\Delta C=50\to \Delta x_{2}=\;\frac{50}{50}=1$

On other hand, $\Delta x_{2}=\;\frac{\beta_{2}}{2\lambda }\;or\;1=\frac{205.3}{2\lambda }\;\to 2\lambda =205.3$

So that mean, $\Delta x_{1}=\frac{\beta_{1}}{2\lambda }=\frac{171}{205.3}\;=0.833$

**Fig. 4:**
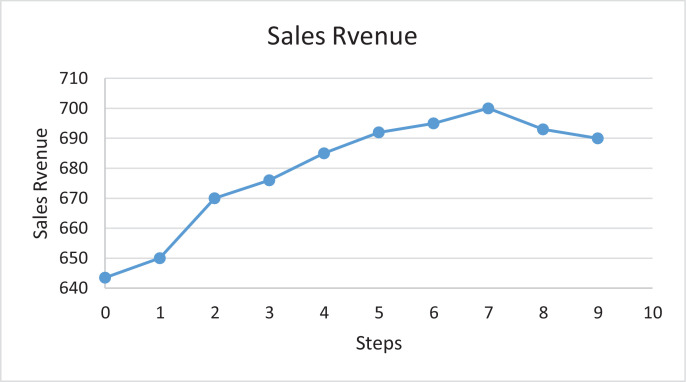
Sales revenue versus steps along the path of steeps ascent.

Points along the path of steepest ascent has been identify and observes the sales revenue at theses point until a decrease in revenue is identified. Fig. 4 plots the revenue at each step along the path of steepest ascent. Increases in response are observed through the seventh steps, but all steps beyond this point result in a decrease in sales revenue. According to that the optimal conditions for the selected marketing strategies that maximize sales revenue are: (593.33 *min*/*week* for *A* and 575 *min*/*week* for *C*). Where the new regression model that fit around the optimal point is:

(7) Y=700+593.33A+575C

### AHP for determining the best marketing strategy

This section is a decision-making model optimization of a lubricant company to identify the best marketing strategy using AHP approach and after using fractional factorial design as screening experiment where we eliminate factors that do not have any significant effect on response (sales revenue). Below are the stages to be followed to come up with best marketing strategy and select the best strategy form the remaining strategies which are in our case strategies *A* and *C*:

1. Identify the criterial for the effective marketing strategies.
2. Criteria weights determination: marketing team input on the criteria weights to be collected and processed.
3. Best marketing selection and ranking**:** the best marketing strategy plan will be chosen in this stage.

Back to the illustrate example, the company's marketing team is asked to set main decision-making criteria of the model along with their respective weights. This exercise is conducted via direct meeting group discussion and below table shows the outcome. Each strategy will be examined using several, multiple criteria that have been provided by the company management along with their relative weights to be taken into account in the evaluation process. These criteria are Cost, Coverage, Interaction and Return-On-Investment (ROI). Table 6 shows criteria and their definitions as stated by marketing team.

**Table 6:** Model decision making criteria definitions.

Criterion	Definition
Cost	Total expenses associated with each type of marketing strategy; including both fixed and variable costs.
Coverage	It is a measure of spread and geographical coverage of receivers that each marketing strategy can reach
Interaction	It is the level of reaction that each marketing strategy can result in after its deployment; How customers are going to perceive and interact with the content of each marketing strategy
Return of Investment	ROI is a quantification of how much return or benefit that each marketing strategy can bring to the overall business once effectively applied. It is a cost: benefit analysis tool

To decide on the selected for a given marketing strategy to the company, it might be quite difficult to find the trade-off between one criterion and the other. For example, what is the coverage level could be achieved under one setup and what is the associated cost increase from one level to another. Such a conflict subjective criteria might be better assessed using a multi criteria decision-making approach such as the Analytic Hierarchy Process (AHP). AHP is commonly used to identify the best alternative marketing strategy.

The procedure that will be followed to select the best marketing strategy between *A* & *C* as follows:

1. Structure the decision-making problem by identify the all criteria and alternative as shown in Table 6.
2. Determine the criteria and the alternative ranking using pairwise using the scale that proposed by the marketing team, shown in Table 7.

**Table 7:** Marketing Team Scale.

Intensity of Importance	Definitions	Explanation
0 to 1	week Importance	Judgement slightly favour one criterion to another
1 to 2	Moderate Importance	Judgement more favour one criterion to another
2 to 3	Strong Importance	Judgement strong favour one criterion to another
3 to 4	Very Strong Importance	Judgement very strong favour one criterion to another
4 to 5	Extreme Importance	Judgement slightly favour one criterion over another is of the highest possible order of affirmation

Relative weights of decision-making criteria with respect to each other is shown in the Table 7. Associated normalized matrix will be calculated also to determine the weighted score of each criterion to be used in related AHP model Table 8.

**Table 8:** Model decision-making criteria and relative scores.

Criteria	Cost	Coverage	Interaction	ROI
Cost	1	1.5	2	0.85
Coverage	0.667	1	0.75	0.35
Interaction	0.5	1.33	1	0.9
ROI	1.18	2.86	1.11	1
**Sum**	***3.34***	***6.69***	***4.86***	***3.10***

Now; the two shortlisted marketing strategies will be evaluated versus each one of the decision criteria in Table 9, Table 10, Table 11, Table 11a, Table 12 and 12 shows the results Table 11a.

**Table 9:** Normalized decision-making criteria matrix with respective weights.

Criteria	Cost	Coverage	Interaction	ROI	Raw Average
**Cost**	30%	22%	41%	27%	***30%***
**Coverage**	20%	15%	15%	11%	***15%***
**Interaction**	15%	20%	21%	29%	***21%***
**ROI**	35%	43%	23%	32%	***33%***
**Sum**	***1.00***	***1.00***	***1.00***	***1.00***	***1.00***

**Table 10:** Evaluating marketing strategies compering to **Cost**.

Cost	TV	Soc. Comm.	Raw Sum	N. Raw Sum
**TV**	1	0.33	1.33	***0.25***
**Soc. Comm.**	3.03	1	4.03	***0.75***
**Sum**	***4.03***	***1.33***	***5.36***	***1.00***

**Table 11:** Evaluating marketing strategies compering to **Coverage**.

Coverage	TV	Soc. Comm.	Raw Sum	N. Raw Sum
**TV**	1	2.50	3.50	**0.71**
**Soc. Comm.**	0.4	1	1.4	**0.29**
**Sum**	**1.40**	**3.50**	**4.90**	**1.00**

**Table 11a:** Evaluating marketing strategies compering to **Interaction**.

Interaction	TV	Soc. Comm.	Raw Sum	N. Raw Sum
**TV**	1	0.35	1.35	**0.26**
**Soc. Comm.**	2.86	1	3.86	**0.74**
**Sum**	**3.86**	**1.35**	**5.21**	**1.00**

**Table 12:** Evaluating marketing strategies compering to **ROI**.

ROI	TV	Soc. Comm.	Raw Sum	N. Raw Sum
**TV**	1	0.25	1.25	***0.20***
**Soc. Comm.**	4	1	5	***0.8***
**Sum**	***5.00***	***1.25***	***6.25***	***1.00***

Now, using the above 4 metrics, an AHP model will be developed for each marketing strategy and the composite weights for the associated two options will be calculated using these formulas where applicable.

(8) $$\text{Weight}\left(\text{TV}\;\text{Advertisement}\right)=\sum_{i=1}^{4}\left(P_{i}P_{TV_{i}}\right)\;$$

(9) $$\text{Weight}\left(\text{Social}\;\text{Networking}\right)=\sum_{i=1}^{4}\left(P_{i}P_{SN_{i}}\right)$$

**Fig. 5:**
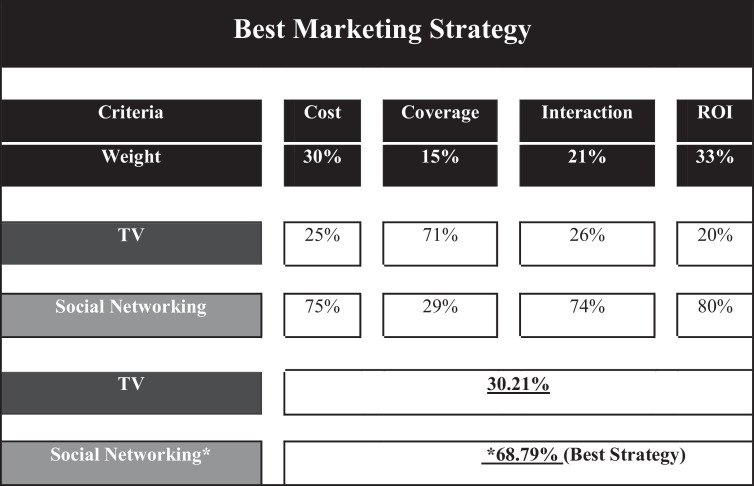
Shows the AHP structure.

Fig. 5 shows the AHP structure and it conclude that the “Social Networking” is best option for the two available strategies.

## Conclusion

The inherent advantages of the fractional factorial design should contribute to increased use of experimental designs by marketing researchers, as it allows the marketing researcher to investigate a large number of marketing strategies and identifying which of these strategies have a significant effect on the response (sales revenue). Combining this advantage with AHP allow decision maker to identify the best market strategy that that maximize the sales revenue and provide the best trade-off between one criterion and the other. To summarize our suggested methodology first finding the most important factors that have an effect on the sales revenue using the Fractional Factorial Design. The, AHP will be used to select the best market strategy. Where the objective is to find which strategies have a significant effect on sales revenue, and then AHP will be used to compare those strategies which have a main effect on the targeted the response and select the best marketing strategy. By using the Fractional factorial design as screening experiment in our example, we conclude that factors *A* and *C* were the ones that have significant effect on the sales revenue. These factors that was derived and further analyzed with the assistance of AHP technique to bring us to our final most important conclusion that factor *C* was the most important factor. In terms of the marketing strategy, it is thus proved that social networking which is factor *C*, is the most pivotal to the company in terms of increasing its revenue from the sales. Fractional factorial design and AHP they should become essential marketing research tools in the future. Where they allow decision maker to investigate a large number of marketing strategy with small number of experimental runs.

## Appedix A

### Obtaining the main effect

To obtain the main effect, for example the factor A main effect: we subtract the sum of the response values where *A* is at its low level from the sum of the response value when A is at its high level and divided it by 4. Table 3 show that *A* is at the high level at location 2,4,6 and 8, and A is at its low level at location 1,3,5 and 7.

Effect of $A=\;\frac{-155+675-410+513-575+1050-750+1020}{4}=342$

We can calculate the remaining main effect by the same methods.

## Appedix B

### Obtaining the sum of squares

To obtain the sum of squares, for example the sum of square for the factor $A=\;\frac{{\left[\left(number\;of\;changes\;in\;level)\times (effect\;of\;A\right)\right]}^{2}}{number\;of\;\text{observations}}=\;\frac{{\left[4\;\times \;342\right]}^{2}}{8}=233928$

We can calculate the remaining main sum of squares by the same methods
